
JOURNAL INFORMATION
==============================
NLM Title Abbreviation: Tex Med J (Austin)
NLM Journal ID: 21130590R

Texas Medical Journal

ISSN: 0892-8495
Publisher: Biomedical Journal Digitization Project

ARTICLE INFORMATION
==============================
PMCID: PMC9522107
Article version: 1
Subjects: Book Notices

Atlas and Essentials of Gynecology

Print publication date: 1897 Nov
Volume: 13
Issue: 5
First page: 259
Last page: 259
License: This work is free of known copyright restrictions. For more information, please see PMC Back Issue Digitization.
License URL: https://creativecommons.org/publicdomain/mark/1.0/

==============================

---

ATLAS AND ESSENTIALS OF GYNECOLOGY. By Dr. Oscar Schaffer, Privatdocent in Obstetrics and Gynecology at the University of Heidelberg, with 173 colored plate illustrations and 54 wood cuts. William Wood & Company, Publishers, 43, 45 and 47, East 10th St., New York, 1897.

This is Volume V, of Wood's Medical Hand Atlases. The book, like the other volumes of this series of hand atlases, is made up largely of illustrations. These are of a very superior character, being both schematic and reproductions from anatomical preparations. In this way the author has illustrated the normal structures and by adding schematic drawings, he has been able to illustrate the variations from the normal through the different stages. This gives the student and the physician the material which personal chemical observations should supply, or serves as the best substitute for actual chemical work and observation. The text is pointed and practical, being as free from repetitions as a work of this kind can be. In the matter of differential diagnosis the author makes use of comparitive tables, thus simplyfing it as much as possible. S. E. H.

---

### Publishers' Notes.

**Fine Location,** black land, railroad town, small competition, will induct purchaser into practice. Residence and office for sale. If you mean business, write for terms, etc. Address, "Juan," care of TEXAS MEDICAL JOURNAL.

---

**I Want a Location,** in a town of 10,000 or more inhabitants. I have a good practice and drug business, also some very desirable property in a live town on a railroad in South Texas, near the coast. Will sell or exchange for a residence or stock of drugs. I have a genuine bargain to offer. Address, Dr. F. Y., care of TEXAS MEDICAL JOURNAL.
